# The association between four neighborhood disadvantage indices and child chronic health classifications

**DOI:** 10.1038/s41390-025-04143-5

**Published:** 2025-05-27

**Authors:** Kahir Jawad, Yana B. Feygin, Michelle Stevenson, Bethany A. Wattles, Jennifer Porter, V. Faye Jones, Deborah Winders Davis

**Affiliations:** 1 Norton Children’s Research Institute affiliated with the University of Louisville School of Medicine, Louisville, KY USA; 2 University of Louisville School of Medicine, Department of Pediatrics, Louisville, KY USA; 3 Norton Children’s Medical Group, Louisville, KY USA; 4 University of Louisville Health Science Center, Office of Health Equity and Engagement, Louisville, KY USA

## Abstract

**Background:**

Neighborhood advantage/disadvantage is a social determinant of health. We aimed to examine the distribution and associations between child chronic health conditions and four commonly used indices.

**Methods:**

Children with outpatient visits and valid addresses (*n* = 115,738) were included and outcomes were categorized as having no chronic disease (N-CD), non-complex chronic disease (NC-CD), and complex chronic disease (C-CD). Four measures of neighborhood characteristics (Child Opportunity Index, Area Deprivation Index, Neighborhood Disadvantage Index, Social Vulnerability Index were calculated from census data. Separate multinomial logistic regression models were used.

**Results:**

The indices’ scores were correlated (*r* = 0.80–0.92). Children in low opportunity or high disadvantage/deprivation/vulnerability neighborhoods were more likely to be diagnosed with C-CD than those in high opportunity or low disadvantage/deprivation/vulnerability neighborhoods. The increased odds ranged from 5% to 39%. The adjusted odds of NC-CD were found to increase by 8–31% as the neighborhood opportunity declined or the disadvantage/deprivation/vulnerability increased, across all indices. The association grew stronger as neighborhood opportunity decreased, or disadvantage/deprivation/vulnerability increased for all four indicators.

**Conclusions:**

Each instrument was associated with medical complexity classifications, but the magnitude of the associations differed slightly. The rationale for choosing a measure of neighborhood characteristics should be based on the study’s aims and population.

**Impact:**

- This study evaluates the associations of four commonly used neighborhood indices with medical complexity classifications. All indices were associated with study outcomes. The Area Disadvantage Index (ADI) and Child Opportunity Index (COI) demonstrated incremental increases in the odds of receiving a classification of complex chronic disease (C-CD) compared to no chronic disease (N-CD) as neighborhood opportunity decreased or the disadvantage/deprivation/vulnerability increased. Being classified with a non-complex chronic disease (NC-CD) compared to N-CD, only the association with the COI increased incrementally at each level of opportunity. Study outcomes and index characteristics must be considered when designing studies.

## Introduction

Understanding health disparities is critical for improving overall health and well-being, particularly in children.^1^ The understanding of how socioeconomic factors affect outcomes and how to address inequities has evolved.

It has long been understood that child health and developmental outcomes occur through complex interactions with the physical and psychosocial environments.^2–5^ These systems models suggest that, beginning in utero and continuing throughout the lifespan, dynamic interactions occur between environmental elements and biology to influence outcomes.^2,3,5–8^ Much early literature focused on the proximal environmental influences such as caregiver-child interactions^9–11^ and the home physical and social environments.^6,12–15^

Recent literature has recognized the importance of neighborhood characteristics on children’s health and development, including the presence of chronic diseases and developmental disabilities.^16–23^ Choi and colleagues found both direct and indirect effects of neighborhood characteristics.^16^ Because of current and historical neighborhood segregation and disparities in neighborhood resources, Black families and other minoritized groups are disproportionately living in disadvantaged neighborhoods.^24–27^ In addition to the associations between environmental influences on child health and development, recent literature has documented the relationship between neighborhood characteristics and pregnancy outcomes such as prematurity and low birth weight.^24,25^ It is important to examine neighborhood quality characteristics as a possible driver that may contribute to disparate health outcomes. Chronic stress caused by racist practices, such as segregation and discrimination, increases exposure to stress hormones and inflammation and predisposes people to chronic diseases.^28,29^ For children, the effects from adverse neighborhood characteristics may be from direct exposures or indirectly through the effects that the environment has on caregivers.^30–32^ With the understanding of the bioecological perspective of the complex array of factors that contribute to child health and development, it is important to consider neighborhood environmental exposures as social determinants of health across generations^33^ and across the lifespan.^34^ A more recent systems model for child health and development is called the ecobiodevelopmental framework.^35^ In this model, the authors urge scientists and clinicians to think beyond biological predispositions that interact with the environment to produce a given outcome.^35^ They suggest that these environmental exposures and early childhood experiences influence the timing and processes around gene activation,^35^ which speaks to the underlying processes responsible for the individual differences in outcomes across the lifespan that have long been identified.

From an epidemiologic perspective, disease risk is influenced by early life exposures including those related to poverty status.^36^ These early exposures can have both early life and long-term effects on health outcomes.^36–41^ Conversely, living in neighborhoods with higher opportunity and/or lower disadvantage/deprivation/vulnerability can have a protective effect against adverse health outcomes.^42^ Childhood is a pivotal phase for later health and developmental outcomes.^8,43^ Environmental and social exposures may have an enduring impact on health outcomes during specific stages of childhood (e.g., infancy, early childhood, adolescence)^16,28,40^ and may be compounded by the dynamic interactions among multiple negative exposures such as those found in under-resourced neighborhoods.^16,28,40,44–46^

Recognizing the importance of neighborhood, researchers have begun to develop tools to measure neighborhood characteristics. In studies of children, four tools have been used most frequently, which rely on census- data to provide a score that can be compared across locations. These tools include The Child Opportunity Index (COI),^47^ Neighborhood Disadvantage Index (NDI),^48^ Area Deprivation Index (ADI),^49^ and Social Vulnerability Index (SVI).^50^ Only the COI was specifically validated with children.^51,52^ However, it is not known whether the remaining three tools are valid for the study of health outcomes in children.^53^

While neighborhood characteristics been previously examined in both the pediatric and adult literature,^37,38,42,54–59^ little is written related about the similarity or differences found in using different neighborhood indices. One study examined both the COI and ADI in relation to adolescent internalizing and externalizing symptoms.^60^ Beyer and colleagues determined that the two measures of neighborhood quality opportunity or deprivation demonstrated different associations with their outcomes and their population.^60^ They concluded that more research is needed to determine if their findings would apply to other physical and mental health outcomes and populations different from children 9–10 years of age.^60^ Another study was published in 2024 that examined the associations between the ADI, SVI, and COI and two pediatric outcomes (attendance at well-child visits and obesity in adolescents).^61^ They concluded that the three measures performed similarly for their two distinct outcomes, but that more research is needed using a variety of outcomes and populations.^61^

In another study, Stephens and colleagues examined the performance of the SVI, ADI, and COI in a large, multi-site study across the U.S. to determine the distribution of the social determinants of health indices for children who undergone surgical procedures.^62^ Rather than examining differences in surgical outcomes, the authors aimed to determine the distribution of the indices scores and the interrater reliability across the different measures.^62^ They found variability between and within hospitals.^62^ Based on their findings, the authors recommend health systems adopt standardized procedures for geocoding to facilitate data-sharing across institutions.^62^ Additionally, they note the differences in the indices’ variables and purposes, and recommend that researchers carefully consider the selection of the index based on the specific aims of the study and the population.^62^ Further, they suggest that using a continuous score instead of the categorical classification may increase the interrater reliability between indices.^62^

Finally, in a study of pediatric surgery outcomes, Chagon and colleagues found the ADI, which was the most studied index, and the COI, which was not well-studied, were the most consistent indicators of neighborhood social determinants of health.^63^ The authors concluded that the COI may be the most useful index since it was developed for the purpose of studying child health and developmental outcomes, but until more data are available for pediatric surgical outcomes, incorporating multiple measures of neighborhood metrics may be beneficial.^63^

Although the literature is limited, it is clear that much more research is needed to determine the “best” metrics moving forward. These studies make it clear that it is important for researchers to have a sound theoretical reason for selecting a specific measure based on their specific study aims and population and the available literature.

In the current study, we aimed to investigate the distribution and associations between the medical complexity classifications of children seen in outpatient settings and the four previously described indices of neighborhood opportunity/deprivation/disadvantage/vulnerability to better understand the neighborhood characteristics associations with child health outcomes and whether the different measures of neighborhood characteristics are interchangeable. Rather than examining a specific individual diagnosis, we opted to use a previously published algorithm that classifies diagnoses by medical complexity (see Methods for additional details).^64,65^

## Methods

### Sample

The sample included 380,266 outpatient visits, which represented 181,887 unique children who were less than 18 years of age. After selecting only those children who had valid home addresses within the KY-IN Louisville Metropolitan Statistical Area (CBSA 31140), the final analytical sample was 115,738 (see Table 1). The cohort was composed of non-Hispanic White children (57.3%), non-Hispanic Black children (25.1%), Hispanic children (9.3%), and children of other race groups (4.7%). Age distribution included 22.4% of children under the age of 2 years, 38.4% ages 3–9 years, and 39.2% ages 10–17 years. Additionally, about half of the children (53.2%) had public insurance, primarily Medicaid; 40.7% had private insurance; 3.5% had self-pay; and 1.7% had other insurance. The majority of children (76.8%) did not have any chronic diseases, whereas 13.5% had non-complex chronic disease and 9.7% had complex chronic disease.

**Table 1:** Analytic sample demographic characteristics.

		Medical Complexity Classification			*p*-value
Variables	Overall	N-CD*	NC-CD	C-CD	
**Overall**	115,738	88,877 (76.8)	15,653 (13.5)	11,208 (9.7)	
**Sex**					< 0.001
Female	57,153 (49.4)	44,995 (50.6)	7174 (45.8)	4984 (44.5)	
Male	58,572 (50.6)	43,869 (49.4)	8479 (54.2)	6224 (55.5)	
**Age group**					< 0.001
0–2 years	25,955 (22.4)	21,024 (23.7)	3152 (20.1)	1779 (15.9)	
3–9 years	44,398 (38.4)	34,501 (38.8)	5870 (37.5)	4027 (35.9)	
10–17 years	45,384 (39.2)	33,351 (37.5)	6631 (42.4)	5402 (48.2)	
**Race/Ethnicity**					< 0.001
White	66,318 (57.3)	50,340 (56.6)	9220 (58.9)	6758 (60.3)	
Black	29,040 (25.1)	21,575 (24.3)	4289 (27.4)	3176 (28.3)	
Hispanic	10,712 (9.3)	8709 (9.8)	1219 (7.8)	784 (7.0)	
Other	5410 (4.7)	4482 (5.0)	565 (3.6)	363 (3.2)	
Unknown	4258 (3.7)	3771 (4.2)	360 (2.3)	127 (1.1)	
**Insurance type**					< 0.001
Public	61,574 (53.2)	9075 (58.7)	6646 (60.0)	45,853 (52.0)	
Private	47,153 (40.7)	5914 (38.3)	4087 (36.9)	37,152 (42.2)	
Self-Pay	1948 (1.7)	253 (1.6)	194 (1.8)	1501 (1.7)	
Other	4000 (3.5)	221 (1.4)	148 (1.3)	3631 (4.1)	

*N-CD no chronic disease, *NC-CD* Non-complex chronic disease, *C-CD* Complex chronic disease. *P*-value of Chi-Square test.

Missing addresses comprised ≤ 4.6% of all children for the outcome categories (C-CD: 4.6%, NC-CD: 2.3%, N-CD: 3.0%). As this falls below the 5% threshold for clinically meaningful bias,^66,67^ and sensitivity analysis was not feasible due to address-dependent predictors (indices), results are presented for patients with complete data. Missing data < 5% for any subgroup is considered negligible as bias from such low rates is typically smaller than sampling error.^66,67^

### Procedures

This cross-sectional secondary data analysis uses the electronic health record (EHR) data from primary care outpatient visits in 2022 at a large healthcare system serving the greater Louisville, Kentucky, and Southern Indiana region, which contains a diverse pediatric population. Children were seen in primary care outpatient settings (family medicine and pediatric), urgent care facilities, and emergency departments. Only children residing in the Louisville/Jefferson County, KY-IN Metropolitan Statistical Area (CBSA 31140) were included. The Metropolitan Statistical Area includes Jefferson County, Kentucky, and Clark and Floyd Counties in southern Indiana.^68^

Race and ethnicity were recategorized as White, Black, Hispanic, Other (American Indian or Alaska Native, Asian, and Native Hawaiian or Other Pacific Islander), and Unknown using the primary variable recorded within the electronic health record (EHR), which includes both race and ethnicity in response options.

Diagnoses were retrieved from encounter diagnoses, past medical histories, problem lists, professional charge transaction diagnoses, final coded diagnoses, and external injury codes. Child health outcomes were categorized using the more conservative version of the Pediatric Medical Complexity Algorithm (PMCA) to identify the cases from the International Classification of Diseases, Tenth Revision, Clinical Modification (ICD-10-CM) codes.^64,65^ While the PMCA authors recommended using three years of data, we used only 2022 due to the effects of COVID-19 on the volume of out-patient visits in 2020 and 2021. Their algorithm categorized a large number of diagnoses into three mutually exclusive groups for children. The groups included children with no chronic disease (N-CD), those with complex chronic diseases (C-CD), and those with non-complex chronic diseases (NC-CD). Examples of each of the classifications include ear infection (N-CD), attention-deficit hyperactivity disorder (NC-CD), developmental delay with a concurrent pulmonary diagnosis (C-CD).^64^ They validated these classifications from a sample of children in an inpatient setting.^64,65^ The algorithm has not been used previously to categorize medical complexity in the out-patient setting. Simon and colleagues reported that, using the least conservative approach of PMCA Version 3.0, there was an 86% sensitivity and 86% specificity in identifying children with C-CD; a 65% sensitivity and 84% specificity for identifying children with NC-CD; and a 77% sensitivity and 93% specificity for identifying children with N-CD.^65^

The exposure variable comprised measures of social disadvantage, including the COI 3.0 (2012 to 2021),^52^ NDI derived from census data for the American Community Survey (ACS) estimate from 2013 to 2017,^69^ ADI v4.0.1 based on 2018 to 2022 ACS 5-Year Data estimates,^49,70^ and SVI 2020, which was extracted from census data for ACS estimate from 2014 to 2018.^71^ These indices scores were assigned using the geocoded patient census tract. The COI total score was normed for the Louisville/Jefferson County, KY-IN Metropolitan Statistical Area (CBSA 31140). The national-normed values for the ADI were used. The SVI and NDI were not normed. The components measured in each index can be found in Supplemental Table S1. Typically, the COI categorizes the opportunity levels by equally distributing the normed total scores into five equal groups (quintiles). The COI, SVI, and NDI were based on census-tract level indicators. The ADI used census-block level indicators. For comparison among the four measures, total scores were converted to quintiles based on our data such that NDI, SVI, and ADI ranged from 1 = least and 5 = most disadvantage/ vulnerability/deprivation/ and COI with 1 = most and 5 = least opportunity. Note that COI measures opportunities whereas the other three indices measure disadvantage, vulnerability, or deprivation.

Spearman correlations between each of the indices were calculated. The Chi-Square test of univariate analysis was utilized to identify potential confounding variables, with a significant *p*-value of < 0.05 (Table 1). Age and sex were included in the multivariable models to control for these confounders. Some variables were not controlled in the analyses due to potential issues with collinearity as follows. Race/ethnicity was not controlled because the SVI contains an estimate of the percentage of individuals who are of minority status. Additionally, minoritized groups are more likely to be of lower income and education status and more likely to live in more disadvantaged/less opportunity neighborhoods. Insurance type was not controlled because all indices have items related to poverty, wealth, education, property value, etc.

Separate multivariable multinomial logistic regression models were used to examine the association between chronic disease levels (C-CD, NC-CD, and N-CD) and each index’s quintile scores (COI, NDI, ADI, and SVI), adjusting for sex and age. The reference group for each model is children with no chronic disease. This project was approved by the University of Louisville Institutional Review Board and the Norton Children’s Research Office.

## Results

### Overall and unadjusted indices’ scores comparisons

**Fig. 1:**
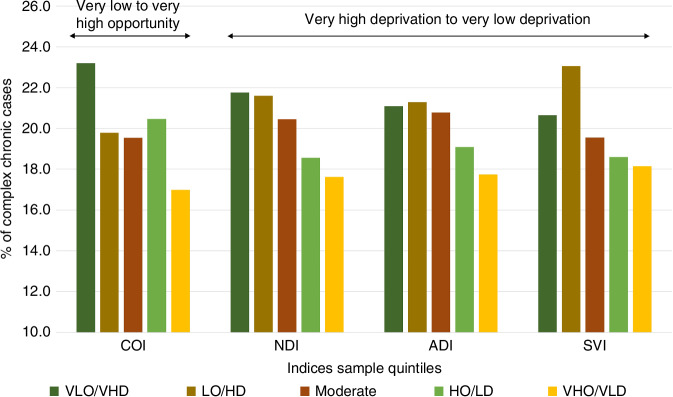
Distribution of cases of children with complex chronic diagnoses by index. COI Child Opportunity Index, NDI Neighborhood Disadvantage Index, ADI Area Deprivation Index, SVI Social Vulnerability Index, VLO/VHD Very Low Opportunity/Very High Deprivation, LO/HD Low Opportunity/High Deprivation, Moderate Opportunity/Moderate Deprivation HO/LD High Opportunity/Low Deprivation, VHO/VLD Very High Opportunity/Very Low Deprivation.

**Fig. 2:**
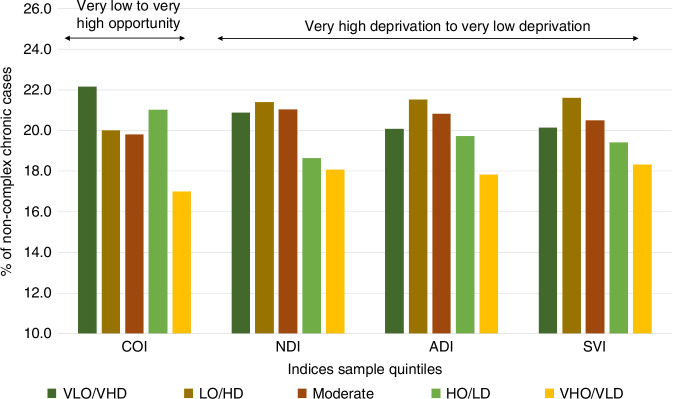
Distribution of cases of children with non-complex chronic diagnoses by index. COI Child Opportunity Index, NDI Neighborhood Disadvantage Index, ADI Area Deprivation Index, SVI Social Vulnerability Index, VLO/VHD Very Low Opportunity/Very High Deprivation, LO/HD Low Opportunity/High Deprivation, Moderate Opportunity/Moderate Deprivation, HO/LD High Opportunity/Low Deprivation, VHO/VLD Very High Opportunity/Very Low Deprivation.

The COI scores correlated with the remaining indices as follows (Table 2): *r* = 0.92 (NDI), *r* = 0.90 (ADI), and *r* = 0.87 (SVI). The smallest Spearman correlation was between the SVI and the ADI (*r* = 0.81). See Figs. 1 and 2 for the unadjusted distribution of the indices’ scores for C-CD and NC-CD diagnoses.

**Table 2:** Relative correlations among indices’ total scores.

	COI	NDI	ADI	SVI
Child Opportunity Index (COI)		0.92	0.90	0.87
Neighborhood Disadvantage Index (NDI)	0.92		0.88	0.85
Area Deprivation Index (ADI)	0.86	0.86		0.80
Social Vulnerability Index (SVI)	0.87	0.85	0.81	

^*^Spearman Correlation Coefficients; All correlations are significant at *p* < 0.001.

### Complex chronic disease compared to no chronic disease

Adjusting for age and sex, multivariable logistic regression (Table 3) found that children were more likely to be diagnosed with a C-CD compared to N-CD based on the neighborhood resource indicators. Recall that the COI measures opportunities, whereas the NDI, ADI, and SVI measure disadvantage, deprivation, or vulnerability.

**Table 3:** Multivariate multinomial logistic regression models for the association between chronic disease levels (C-CD, NC-CD, and with N-CD) and indices’ quintiles: COI, NDI, ADI, and SVI.

	Complex chronic (C-CD) vs no chronic disease (N-CD)			
	COI	NDI	ADI	SVI
	aOR (95% CI)	aOR (95% CI)	aOR (95% CI)	aOR (95% CI)
Q1: Very high/Very low (highest quality)	ref			
Q2: High/Low	1.14 (1.07–1.22)	1.13 (1.06–1.21)	1.15 (1.08–1.23)	1.05 (0.99–1.12)^†^
Q3: Moderate/Moderate	1.22 (1.14–1.31)	1.20 (1.12–1.28)	1.25 (1.17–1.33)	1.14 (1.07–1.22)
Q4: Low/High	1.30 (1.22–1.39)	1.33 (1.25–1.42)	1.33 (1.24–1.41)	1.34 (1.26–1.42)
Q5: Very low/Very high (lowest quality)	1.39 (1.30–1.48)	1.32 (1.24–1.41)	1.38 (1.29–1.47)	1.27 (1.19–1.35)
	**Non-complex chronic (NC-CD) vs no chronic disease (N-CD)**			
	**COI**	**NDI**	**ADI**	**SVI**
	**aOR (95% CI)**	**aOR (95% CI)**	**aOR (95% CI)**	**aOR (95% CI)**
Q1: Very high/Very low (highest quality)	ref			
Q2: High/Low	1.16 (1.10–1.23)	1.10 (1.04–1.16)	1.18 (1.11–1.24)	1.08 (1.02–1.14)
Q3: Moderate/Moderate	1.22 (1.16–1.30)	1.19 (1.13–1.26)	1.22 (1.16–1.29)	1.17 (1.11–1.23)
Q4: Low/High	1.30 (1.23–1.37)	1.27 (1.20–1.34)	1.31 (1.24–1.38)	1.22 (1.16–1.29)
Q5: Very low/Very high (lowest quality)	1.31 (1.24–1.39)	1.22 (1.16–1.29)	1.29 (1.22–1.36)	1.21 (1.15–1.28)

*aOR adjusted odds ratio, *CI* confidence interval, *COI* Child Opportunity Index 3.0, *NDI* Neighborhood Deprivation Index, *ADI* Area Disadvantage Index v4.0, *SVI* Social Vulnerability Index; adjusted for age and sex.

All associations are significant at *p* < 0.05 except one aOR, ^†^non-significant *p*-value.

The likelihood of being diagnosed with a C-CD, as compared to N-CD, increased gradually from Very High opportunity to Very Low opportunity using the COI (Table 3). In other words, living in the lowest opportunity neighborhood was associated with a greater chance of receiving a C-CD diagnosis classification compared to N-CD than living in the highest opportunity. Using the NDI, ADI, or SVI, those living in the Very Low deprivation/disadvantage/vulnerability neighborhoods had the lowest odds of receiving a C-CD classification compared to N-CD than those living in the lowest deprivation/disadvantage/vulnerability neighborhood. For the NDI, compared to the Very Low deprivation, the aORs ranged from 1.13 (Low deprivation) to 1.32 (Very High deprivation). For the ADI, the aORs ranged from 1.15 (Low disadvantage) to 1.38 (Very High disadvantage). For SVI, the aORs ranged from 1.05 (Low vulnerability) to 1.27 (High vulnerability). In summary, children living in the Very Low opportunity or Very High deprivation/disadvantage/vulnerability neighborhoods had the greatest odds of receiving a C-CD diagnosis compared to N-CD, and the odds decreased as the neighborhood opportunity increased or the deprivation/disadvantage/vulnerability decreased (See Table 3). All aORs were statistically significant except for one, which was the second quintile of the SVI (1.05 (0.99–1.12)).

### Non-complex chronic vs no chronic disease

Similarly, though to a lesser extent, the adjusted odds of being diagnosed with a NC-CD compared to a healthy N-CD increased progressively from the highest opportunity or lowest deprivation/disadvantage/vulnerability to the lowest opportunity or highest deprivation/disadvantage/vulnerability neighborhood for all four indices. The aOR of receiving a NC-CD diagnosis compared to N-CD diagnosis ranged from 1.16 to 1.31 for COI, 1.10 to 1.22 for NDI, 1.18 to 1.29 for ADI, and 1.08 to 1.21 for SVI when comparing each quintile to the highest quality. All aORs were statistically significant.

### Overall comparisons of indices

Model fit was tested statistically using the Akaike Information Criterion (AIC). A lower AIC is indicative of a better fit to the data. The ADI model showed a lower AIC than other models, indicating the best fit among all four models. Due to collinearity, all indices cannot be simultaneously entered into one model. Therefore, the AIC was used the evaluate model fit.

## Discussion

The current study provides new information on the association between four different indices of neighborhood quality and diagnostic classifications. Each index appeared to distinguish classifications of complex chronic and non-complex chronic from no chronic disease. The ADI and COI demonstrated incremental increases in the odds of receiving a C-CD classification compared to N-CD classification as neighborhood indicators went from higher opportunity or lower deprivation/disadvantage/vulnerability to lower opportunity or higher deprivation/disadvantage/vulnerability. There was variability across indices such that no measure was consistently different from the others at every level of neighborhood opportunity or deprivation/disadvantage/vulnerability. When looking at the odds of being classified with a NC-CD compared to N-CD, only the association with the COI increased incrementally at each level of opportunity when going from the highest to the lowest neighborhood opportunity or lowest to highest deprivation/disadvantage/vulnerability. The COI and ADI performed similarly but, again, none of the indices consistently differed at each level of neighborhood opportunity or deprivation/disadvantage/vulnerability. These findings are in accordance with other studies on health equity that have shown that neighborhood quality is associated with adverse outcomes.^24,25,40,72^

In a recent systematic comparison, Lou and colleagues similarly found variations between different indices of neighborhood characteristics for a variety of outcomes in adults.^73^ They concluded that the ADI and the COI were the most robust measures across multiple health outcomes.^73^ Two previous studies in pediatrics examined similar neighborhood indices.^61,74^ One study found that the COI and ADI had different associations with their two outcomes (adolescent internalizing and externalizing symptoms).^74^ Another study examined the associations between the ADI, SVI, and COI and attendance at well-child visits and adolescent obesity.^61^ They found that the three measures performed similarly for their outcomes. The authors of both papers concluded that more research is needed using a variety of outcomes and populations to better understand how these indices perform in different contexts.^61,74^

Including measures of neighborhood characteristics in research is important as structural racism practices such as “redlining”^75^ and other systemic forms of segregation and discrimination resulted in impoverished neighborhoods that continue to be associated with lower incomes, lower property values, lower life expectancies, and poorer health outcomes.^24,25,72,75–77^ More research is needed, especially related to child health outcomes, to better understand the role of neighborhoods as a mechanism through which child health disparities occur. Importantly, to develop interventions to promote optimal health for all children, especially the most vulnerable, we must first have a better knowledge of elements that may be protective in the face of disparities in neighborhood resources.

Although the study provides new information, there are limitations. First, the data for these analyses came from a single healthcare system; therefore, generalizability to other locations may be limited. That said, the system consists of both academic pediatric practices as well as community family medicine and pediatric practices. Therefore, well children without chronic diseases should have been captured during well-child checks. Second, the PMCA was previously applied to inpatient discharge data. The algorithm has not been applied to outpatient data. The authors of the PMCA recommended using three years of data, but we used only 2022 due to the effects of COVID-19 on the volume of out-patient visits in 2020 and 2021. The use of a single year of data could have an effect on the classification. However, data were captured from multiple places within the EHR such that diagnoses from previous health care visits were included. Third, electronic medical record data were used. There are inherent limitations for all secondary data analyses, especially administrative data such as electronic medical record data. Fourth, the sample consisted of children who had at least one outpatient visit to the healthcare system during the year. We cannot account for those not engaged in the healthcare system. Lastly, just under 5% of the sample was missing an address, so they were excluded. Although there is a potential for bias since more vulnerable children may be more likely to not have a stable home address, there is no way to assess bias as an address is required for each of the indices. However, since the missing data are less than 5% in all outcome classifications, the bias can be considered negligible as the rates are typically smaller that sampling error.^66,67^

Despite the limitations, the study has implications for future research. Past research has often failed to explain the rationale for choosing a neighborhood index. Careful consideration of the chosen measure and a clearly written rationale within the methods would strengthen future studies. For this study, all indices were associated with our study outcomes related to medical complexity disease classifications. However, the indices may perform differently for different outcomes and different populations. One consideration for the COI is that it was designed with a focus on children, and it has some important qualities to consider when designing studies about child health and developmental outcomes. The index can be used as an overall score or with any or all of the three domains (educational opportunities, health and environmental opportunities, and social and economic opportunities).^47,51^ Additionally, COI 3.0 has identified 14 subdomains to allow for more granular exploration of factors that may be associated with various child health and developmental outcomes.^78^ One major difference between the COI and the ADI, SVI, and NDI is the inclusion of health-related factors such as proximity to health care facilities, retail healthy food environment index, proximity to toxic waste release sites, volume of nearby toxic waste release, proximity to parks and open spaces, and housing vacancy rate (See Supplemental Table S1). For studies of child development, educational opportunities such as proximity to licensed early childhood education centers and high-quality early childhood education centers, which are captured with the COI, may be critically important.

The COI is normed for the U.S. general population as well as a number of city/county locations. There is a growing body of literature to support the use of the COI in various pediatric populations and conditions.^79–84^ However, continued vigilance is needed to operationalize the measurement of neighborhood characteristics based on the study aims, population, and outcomes of interest. The measures were uniquely developed and validated for specific purposes and should be used based on sound rationale.

Some considerations for future research are the inclusion of children from different geographic locations, the use of prospective longitudinal methods, children with specific mental and physical health diagnoses, and the study of the assessment of differences by race/ethnicity. Prospective longitudinal methods would allow for the analysis of temporal effects and to capture geographic mobility across time. Since minoritized families are more likely to live in lower-resourced neighborhoods^25,57,85^ and to experience higher rates of many mental and physical health problem,^25,57,86–88^ understanding the mechanisms of action for negative associations between neighborhood characteristics and health diagnoses is critical to developing policies and practices to support optimal health for all children.

In the current study, we found that all indices were associated with our study outcomes, but with some variation in magnitude. However, these associations apply only to the medical complexity classifications. Considerations for future studies using neighborhood indices include selecting the measures based on a study’s outcomes and target populations. For example, if a study focuses on health (e.g., mental health, physical health), neighborhood indices that measure factors like air quality, green space, noise levels, access to healthcare facilities, and walkability would be preferable. If a study involves social or economic outcomes the researcher may want to focus on indices that measure safety like neighborhood crime rates or violent crime index, education access and quality such as school quality ratings and/or school dropout rates, and economic opportunity (e.g., employment rates, income levels).

Other considerations include characteristics of the population of interest such as age, socioeconomic status, and racial, ethnic, and cultural backgrounds. Additionally, the researcher should consider the availability of relevant and reliable data for their specific location and whether those data are available at the geographic scale necessary to answer their research question (census tracts, zip codes, etc.).

Since the COI was designed specifically for use with children, more research using the total score as well as the three domains and 14 subdomains for relevant outcomes is needed. Again, to inform policy changes, study designs that focus on the mechanisms through which these environmental factors impact child outcomes are critically important.

Continued diligence in identifying mechanisms of action for disparities is necessary to optimize health and developmental outcomes for all children. Proper use of neighborhood indices has the potential to contribute to our understanding of the role of neighborhood in creating child health and developmental disparities and to target the use of our limited resources on the drivers of disparities. For example, in a study of aging, Simons and colleagues found that social adversity and discrimination were better predictors of accelerated aging, and that personal health behaviors were not related to the rate of aging.^89^ However, despite finding no effect of individual health behaviors on the aging process, much literature focuses on individual interventions.^89^ Simons and colleagues suggest that individual solutions cannot eliminate the health disparities that are the result of social structures that create poor quality neighborhoods and barriers to healthier lifestyles.^89^ Understanding how these structures influence child health and developmental outcomes could inform the development of interventions earlier to minimize the negative effects. A body of literature is emerging that supports racism and discrimination as social determinants of child health and development.^90–94^ Focusing policies to minimize disparities in neighborhood resources has potential to improve child health and development in ways that individual-level interventions cannot.

## Supplementary information

Supplemental table

## Data Availability

Data are not publicly available due to a data use agreement. A de-identified dataset may be available upon request.
