# Supplementary material for: The association between four neighborhood disadvantage indices and child chronic health classifications
Source: Pediatr Res. 2025 May 27;99(1):292–300. doi: 10.1038/s41390-025-04143-5 (PMC12920081; doi:10.1038/s41390-025-04143-5)
Supplement: Supplementary file 1 — Supplemental table [file 41390_2025_4143_MOESM1_ESM.pdf]

Supplemental Table S1- Description of all four indices domains and variables

Child Opportunity Index (COI)*	
**Domain**	**Variables**
Educational opportunities	School poverty rate (eligibility for free or reduced-price lunch)
	Student math proficiency level
	Student reading proficiency level
	Proximity to licensed early childhood education centers
	Proximity to high-quality early childhood education centers
	Early childhood education participation
	High school graduation rate
	Adult educational attainment
Health and environmental opportunities	Proximity to health care facilities
	Retail healthy food environment index
	Proximity to toxic waste release sites
	Volume of nearby toxic waste release
	Proximity to parks and open spaces
	Housing vacancy rate
Social and economic opportunities	Foreclosure rate
	Poverty rate
	Unemployment rate
	Public assistance rate
	Proximity to employment

* The Child Opportunity Index 3.0 (COI) is a composite index of children's neighborhood opportunity that contains data for every neighborhood (census tract 2020) in the United States from every year for 2012 through 2021. It is comprised of 44 indicators in three domains (education, health and environment, and social and economic) and 14 subdomains. retrieved from https://data.diversitydatakids.org/dataset/coi30-2020-tracts-child-opportunity-index-3-0-database--2020-census-tracts?_external=True on Jun 23 2024.
This study categorizes the variable (r_COI_met; Metro normed Child Opportunity Scores (from 1 to 100)) into five equal quintiles.

Neighborhood Deprivation Index (NDI)*	
**Domain**	**Variables**
Wealth & Income	Percent of households receiving public assistance
	Median home value (dollars)
	Percent of families with incomes below the poverty level
Housing Conditions	Percent of households that are female headed with any children under 18
	Percent of housing units that are owner occupied
	Percent of households without a telephone
	Percent of households without complete plumbing facilities
Education	Percent with a high school degree or higher
	Percent with a college degree or higher
Occupation	Percent in a management, business, science, or arts occupation
	Percent unemployed

*A Neighborhood Deprivation Index (NDI) for each Census tract in the U.S. was created using factor analysis to identify key variables from 13 measures in the following dimensions of socioeconomic (SES) status: wealth and income, education, occupation, and housing conditions. These 13 variables were obtained from the Census Bureau's 5-year American Community Survey (ACS) data for 2013-2017. This study categorizes the variable (NDI) into five equal quintiles

Area Deprivation Index (ADI)*	
**Domain**	**Variables**
Education	% Population aged 25 years or older with less than 9 years of education
	% Population aged 25 years or older with at least a high school diploma
	% Employed population aged 16 years or older in white-collar occupations
Income/employment	Median family income in US dollars
	Income disparity
	% Families below federal poverty level
	% Population below 150% of federal poverty level
	% Civilian labor force population aged 16 years and older who are unemployed
Housing	Median home value in US dollars
	Median gross rent in US dollars
	Median monthly mortgage in US dollars
	% Owner-occupied housing units
	% Occupied housing units without complete plumbing
Household characteristics	% Single-parent households with children younger than 18
	% Households without a motor vehicle
	% Households without a telephone
	% Households with more than 1 person per room

*The Area Deprivation Index (ADI) is based on a measure created by the Health Resources & Services Administration (HRSA) over three decades ago, and has since been refined, adapted, and validated to the Census block group neighborhood level by Amy Kind, MD, PhD and her research team at the University of Wisconsin-Madison. For more details: https://www.neighborhoodatlas.medicine.wisc.edu
This study categorizes the variable (ADI_NATRANK (from 1 to 100)) into five equal quintiles.

Social Vulnerability Index (SVI)	
**Domain**	**Variables**
Socioeconomic Status	Percentile Percentage of persons below poverty estimate
	Percentile Percentage of civilian (age 16+) unemployed estimate
	Percentile per capita income estimate
	Percentile Percentage of persons with no high school diploma (age 25+) estimate
Household Composition & Disability	Percentile percentage of persons aged 65 and older estimate
	Percentile percentage of persons aged 17 and younger estimate
	Percentile percentage of civilian noninstitutionalized population with a disability estimate
	Percentile percentage of single parent households with children under 18 estimates
Minority Status & Language	Percentile percentage minority (all persons except white, non- Hispanic) estimate
	Percentile percentage of persons (age 5+) who speak English "less than well" estimate
Housing Type & Transportation	Percentile percentage housing in structures with 10 or more units estimate
	Percentile percentage mobile homes estimate
	Percentile percentage households with more people than rooms estimate
	Percentile percentage households with no vehicle available estimate
	Percentile percentage of persons in group quarters estimate

*The Centers for Disease Control and Prevention and Agency for Toxic Substances and Disease Registry Social Vulnerability Index (SVI) is a place-based index, database, and mapping application designed to identify and quantify communities experiencing social vulnerability. For more details: https://www.atsdr.cdc.gov/placeandhealth/svi/index.html
This study categorizes the variable (RPL_THEMES (from 0 to 1)) into five equal quintiles.
